# Culture Moderates the Relationship Between Emotional Fit and Collective Aspects of Well-Being

**DOI:** 10.3389/fpsyg.2018.01509

**Published:** 2018-08-24

**Authors:** Sinhae Cho, Natalia Van Doren, Mark R. Minnick, Daniel N. Albohn, Reginald B. Adams, José A. Soto

**Affiliations:** Department of Psychology, Pennsylvania State University, University Park, PA, United States

**Keywords:** emotional fit, cultural fit, well-being, collective self-esteem, group identification, collective identity, culture

## Abstract

The present study examined how emotional fit with culture – the degree of similarity between an individual’ emotional response to the emotional response of others from the same culture – relates to well-being in a sample of Asian American and European American college students. Using a profile correlation method, we calculated three types of emotional fit based on self-reported emotions, facial expressions, and physiological responses. We then examined the relationships between emotional fit and individual well-being (depression, life satisfaction) as well as collective aspects of well-being, namely collective self-esteem (one’s evaluation of one’s cultural group) and identification with one’s group. The results revealed that self-report emotional fit was associated with greater individual well-being across cultures. In contrast, culture moderated the relationship between self-report emotional fit and collective self-esteem, such that emotional fit predicted greater collective self-esteem in Asian Americans, but not in European Americans. Behavioral emotional fit was unrelated to well-being. There was a marginally significant cultural moderation in the relationship between physiological emotional fit in a strong emotional situation and group identification. Specifically, physiological emotional fit predicted greater group identification in Asian Americans, but not in European Americans. However, this finding disappeared after a Bonferroni correction. The current finding extends previous research by showing that, while emotional fit may be closely related to individual aspects of well-being across cultures, the influence of emotional fit on collective aspects of well-being may be unique to cultures that emphasize interdependence and social harmony, and thus being in alignment with other members of the group.

## Introduction

While early research has conceptualized emotions as largely intrapersonal experiences that take place within individuals, emotions are also social (Parkinson, 1996) and emerge from dynamic interactions between individuals and their social environment (Campos et al., 1989; Lazarus, 1991; Mesquita, 2010). Because the social environment is culturally constructed, the interaction between individuals and their social environment can lead to variations in emotional experiences across cultures (Markus and Kitayama, 1991; Mesquita and Frijda, 1992). At one level, this cultural difference in emotions can manifest in the form of varying preferences for and prevalence of particular types of emotions (Eid and Diener, 2001; Kitayama et al., 2006; Tsai et al., 2006). At another level, culture may have a broader impact on how we understand emotions. For example, in Western culture, where the autonomy and boundaries of each individual are highlighted, emotions are seen as psychological events that occur within an individual. In contrast, in East Asian culture, where the mutual interdependence between individuals is highlighted, emotions are viewed as social and moral processes (Masuda et al., 2008; Mesquita, 2010).

Variability across cultures in the emphasis of the social context in emotions suggests that the degree to which the emotional norms of one’s culture affect individuals may also vary as a function of culture. Recently, studies have investigated how the degree of similarity or coherence between an individual’s own emotional pattern and the emotional patterns of others in the same environment – emotional fit with culture – predicts well-being (De Leersnyder et al., 2014, 2015). These studies demonstrate that emotional fit is important for individuals’ well-being across cultures, but that culture may also have a critical moderating role in this relationship. Building on this research, the present study examines the role of culture in how emotional fit relates to well-being in Asian American and European American college students. Specifically, we focus on collective aspects of well-being (i.e., positive collective identity as indexed via collective self-esteem and identification with one’s group) in addition to examining individual aspects of well-being (i.e., psychological well-being indexed via life satisfaction and depression). We also expand on previous research by exploring how these different aspects of well-being relate to multiple indices of emotional fit derived from self-report, behavioral, and physiological markers of emotions.

### Emotional Fit and Individual Well-Being

There is growing evidence to support the notion that experiencing similar patterns of emotions to others within the same culture is important for individual well-being (De Leersnyder et al., 2014, 2015). In a series of studies, De Leersnyder and colleagues directly measured, rather than inferred, emotional fit with culture by using a profile correlation approach – correlating each individual’s pattern of emotions in response to different situations with the average emotional pattern of the group. They then assessed the association between emotional fit and well-being in three different cultures (United States, Belgium, and Korea). Their results revealed that having higher emotional fit in relationship-focused situations (i.e., situation that involved relationship with others) was associated with greater relational well-being (i.e., having good interpersonal relationships) across all cultures (De Leersnyder et al., 2014). Emotional fit also predicted psychological well-being across cultures, although the specific contexts in which emotional fit mattered varied depending on culture (i.e., relationship-focused situations in Korea, and self-focused situations in the United States; De Leersnyder et al., 2015). These findings suggest that although there may be some cultural variability in how emotional fit relates to individual well-being, emotional fit is generally important for well-being at some basic level across cultures.

Evidence from research examining cultural norms and well-being further support this point. Being in alignment with the normative practice of one’s own culture is important for individuals’ adjustment and well-being (Oishi and Diener, 2001; Kitayama et al., 2010). While the cultural mandates for well-being may vary across cultures, it is universal for people to achieve well-being through actualizing their respective cultural mandates. For example, actualizing values of autonomy and personal control would lead to well-being in Western culture, whereas actualization of the values of interdependence and relational harmony leads to well-being in East Asian culture. In a cross-cultural study comparing Americans and Japanese, it was indeed shown that personal control was the strongest predictor of well-being in the United States, but the absence of relational strain was most predictive of well-being in Japan (Kitayama et al., 2010). Similarly, attaining relational goals, and thus actualizing cultural mandates of interdependent culture was closely associated with well-being among Asian Americans and Japanese, but not among European Americans. In contrast, attaining independent goals was related to well-being in European Americans but not among Asian Americans or Japanese (Oishi and Diener, 2001). In sum, these studies suggest that fitting with norms of cultures is important for achieving individual well-being regardless of one’s cultural orientation, even if those norms vary from culture to culture.

### Emotional Fit and Collective Identity

Parallel to the individualistic focus on the conceptualization and study of emotions as an intra-individual phenomenon, studies of well-being and adjustment have also traditionally emphasized the individualistic, personal aspects of well-being (e.g., personal self-esteem). Yet, individuals’ well-being and adjustment are also closely related to the collectivistic aspects of the self. For example, having a positive collective identity, indexed via collective self-esteem – the tendency to have a positive view about one’s group identity – has been found to be associated with psychological well-being (Crocker et al., 1994). This relationship was evident especially in Asians (vs. European Americans) even after controlling for the effect of personal self-esteem, reflecting the greater emphasis on the group and group experiences in Asian culture. Given that collective identity may be an important index of well-being that complements the index of individualistic well-being, the current study focuses on the relationship between emotional fit and collective identity (i.e., collective self-esteem and identification with one’s group) in addition to the individualistic indices frequently used in studies of well-being (i.e., life satisfaction and depression).

Previous research suggests that the experience of shared emotions with group members is important for constructing a positive group identity (Livingstone et al., 2011; Páez et al., 2015). For instance, Páez et al. (2015) found that perception of emotional synchrony while participating in collective gatherings (i.e., folkloric marches and protest demonstrations) led to greater collective self-esteem and increased identity fusion with the group. Similarly, in a laboratory study that employed an experimental manipulation of emotional fit with pre-existing and arbitrary groups, participants with increased emotional fit with the group indicated greater identification with the group, even when the group was created arbitrarily and carried no real meaning (Livingstone et al., 2011). On the other hand, some research also suggests that group identification may lead to shared emotional experience as well (Weisbuch and Ambady, 2008; Tanghe et al., 2010). For example, Tanghe et al. (2010) showed that increasing group identification through a laboratory manipulation led to greater similarity in emotional experience among group members.

While these studies suggest that emotional fit may be generally important for achieving positive collective identity (higher collective self-esteem and stronger identification with a group), studies have not yet examined cultural differences in how emotional fit relates to collective identity. However, cross-cultural theorists have long discussed how one’s sense of self is closely tied to others in interdependent cultures, whereas it is construed more independently in independent cultures (Markus and Kitayama, 1991). Thus, it follows that collective identity should be affected by the degree of shared experiences with group members to a greater extent in interdependent cultures than in independent cultures, making the link between shared emotional experiences (i.e., emotional fit) and collective self-esteem and group identification especially pronounced in East Asian culture.

### Broadening the Assessment of Emotional Fit

Previous work on emotional fit has primarily focused on similarity in the patterns of *subjective* (i.e., self-reported) emotional responses between an individual and a reference group. The current study takes a multi-method approach to the assessment of emotions, and therefore to the measurement of emotional fit. We see emotions as a multi-componential construct that comprise subjective, behavioral, and physiological responses. Although some theories of emotion assume response coherence across the various components of an emotional response (e.g., Ekman, 1992; Levenson, 1994), empirical support for the response system coherence is largely inconsistent. Recently, a dual-process perspective on emotion response coherence has been proposed to reconcile this inconsistency (Evers et al., 2014). This framework suggests two relatively independent emotion systems: one automatic system that is relatively unconscious and fast (e.g., physiological response) and another reflective system that is relatively conscious and deliberate (e.g., subjective and behavioral responses). While the two emotion systems are thought to work together to promote adaptive behaviors (Baumeister et al., 2007), the response coherence between the two systems tends to be weak or non-existent in contrast to the coherence evident between varying indicators within each system (Evers et al., 2014). This lack of coherence suggests that emotional fit in one of these response domains may not necessarily be associated with emotional fit in another.

The potential variability in emotional fit across emotional response domains (subjective, behavioral, and physiological) may also carry important implications for how emotional fit plays out in different cultures. According to Levenson’s biocultural model of emotion (Levenson, 2003; Levenson et al., 2007), self-reports of subjective experience are highly susceptible to cultural influences, facial expressions are somewhat susceptible to cultural influences, and physiological response tendencies are relatively uninfluenced by culture. Because self-reports and behavioral expressions of emotions are visible and can directly influence social interactions, these may need to be modulated according to cultural norms more so than physiology. Therefore, emotional fit with culture may be more likely among subjective and behavioral response domains than in physiological responses. These ideas have yet to be examined empirically, however, because of the narrow interpretation of emotional fit in the literature.

Given the complexity of emotional experiences and varying cultural influence on emotion systems, the current study sought to broaden the concept of emotional fit by using assessments of both automatic and reflective emotion systems. We assessed individuals’ subjective (self-report), behavioral (facial expression), and physiological (cardiovascular) responses to emotional stimuli to determine indices of self-reported, behavioral, and physiological emotional fit. Self-report measures of emotion are thought to capture the reflective emotion system, and physiological arousal associated with an emotional response are believed to reflect the automatic system. Facial expressions likely represent a combination of both reflective and automatic processes given evidence for both universal and culturally variable components of facial expressions (Levenson et al., 2007).

### The Present Study

The present study examines the associations between emotional fit and individual and collective aspects of well-being among a sample of East Asians/Asian Americans (henceforth, Asian Americans) and European Americans. Because we were interested in capturing representatives of two broad cultural groups whose traditional values regarding self and relationship are quite different, we employed stringent criteria that made use of behavioral markers of cultural orientation, family origin criteria, and self-identification to operationalize our cultural groups. These criteria are outlined in the methods and are meant to increase the likelihood that the cultural groups studied reflect the traditional norms and values associated with their respective cultural heritages, which include differential emphasis on social contexts in determining well-being.

In measuring the construct of emotional fit, we used a method from De Leersnyder et al. (2014) that considers the *patterns* of emotional experience in relation to those of the same cultural group. Here, we measured emotional fit objectively by taking the correlation between the individual’s emotional pattern and the average pattern of the group (see the section “Materials and Methods” for details). Thus, rather than reflecting a subjective awareness of one’s fit with one’s cultural group, this conceptualization of emotional fit reflects an objective measure of normative emotional responding. While it is possible that subjective awareness of emotional fit may also provide valuable information about the relationship between emotional fit and well-being, the subjective measure of fit may be susceptible to demand characteristics. On the other hand, the objective measure of emotional fit allowed us to explore the direct link between normative emotional responding and well-being while separating the effect of demand characteristics (De Leersnyder et al., 2014).

To test our research question, we reanalyzed data originally collected as part of a large multi-method project investigating cultural difference in emotional reactivity and regulation. Results of the rest of the experiment are reported elsewhere (Soto et al., 2016). Although these data were not designed for the purposes of analyzing emotional fit, and therefore was largely a convenience data set, it did afford several opportunities to advance the emotional fit work and expand it in novel ways. This was an experimental study that collected self-report, behavioral (facial expression), and physiological responses to varying emotional stimuli, with participants being asked to regulate their emotional behavior (i.e., suppress or amplify) for a subset of the trials. Assessing various components of emotions in this study allowed us to explore emotional fit at multiple levels and in multiple ways. Thus, in the present study we examined emotional fit based on self-reported emotions (henceforth, self-report emotional fit) as well as emotional fit based on behavioral and physiological responses (behavioral emotional fit and physiological emotional fit, respectively). We were also able to look at emotional fit in different emotional response contexts (baseline emotional responding, in response to neutral stimuli, and in response to negative stimuli).

We tested two primary hypotheses in the present study. Based on previous studies supporting the relationship of individual well-being with self-report emotional fit (De Leersnyder et al., 2015) and with actualization of cultural norm (Oishi and Diener, 2001) across cultures, we hypothesized that self-report emotional fit would be associated with greater individual well-being (as indexed via increased life satisfaction and lower depression) in both Asian Americans and European Americans. In addition, we hypothesized that self-report emotional fit would be associated with more positive collective identity (as indexed via greater collective self-esteem and increased identification with group) based on previous evidence supporting this link (Livingstone et al., 2011; Páez et al., 2015). Importantly, we also predicted that culture would moderate this relationship, because in many East Asian cultures the self is construed in relation to others (Markus and Kitayama, 1991), and thus, being in alignment with others may have a greater impact on the collective identity of Asian Americans than European Americans. Thus, we expect that the positive association between self-report emotional fit and collective identity will be stronger in Asian Americans than in European Americans. In addition to testing these hypotheses, we conducted a series of exploratory analyses to test whether or not the hypothesized patterns of results for self-report emotional fit would replicate with the behavioral and physiological emotional fit indices.

Lastly, the design of the original experiment allowed us to investigate emotional fit across different emotional contexts. It is becoming increasingly important to recognize the contextualized nature of emotions (Scherer, 2009; Izard, 2010; Aldao, 2013). Emotion researchers have called for increased attention to the cultural and social context of emotions at the collective level in order to enhance our understanding of emotions as a whole (Goldenberg et al., 2017). This view also calls for the need to understand emotions in the context of particular emotional situations. This is because cultural differences in emotional experience occur in part as a function of varying situation selections across cultures (De Leersnyder et al., 2013). This means that findings from cultural investigation of emotions may vary depending on what emotional *situation* has been examined in the study. This highlights the importance of studying and understanding emotions in relation to particular emotional situations. Thus, in this study, we examined participants’ emotional fit at three different experimental time points: prior to the introduction of any emotional stimuli (Time 0), in response to a neutral film (Time 1), and in response to a disgust-inducing film (Time 2). Previous studies on emotional fit examined mostly participants’ broad emotional patterns in a particular environment (e.g., family or work settings; De Leersnyder et al., 2015). We thought that this approach would be most comparable to self-report emotional fit at baseline (Time 0) where participants were in the same setting, prior to presentation of any laboratory emotional stimulus. Thus, our primary hypotheses relating to self-report emotional fit and well-being are specific to measurement of emotional fit at Time 0. However, we also explored whether or not any of the findings observed at Time 0 are also seen at Times 1 and 2 when specific emotional stimuli are introduced.

## Materials and Methods

### Participants

The final sample consisted of 127 undergraduate students recruited at a large university in the northeastern United States. Fifty two participants (29 females; 23 males) were identified as East Asians or Asian Americans (referred to as Asian Americans throughout the paper) and 75 participants (49 females; 25 males; 1 missing gender information) were identified as European Americans. Among the total of 127 participants, the age information was missing for 24 participants due to experimenter errors. The average age of the remaining 103 participants was 19.50 (*SD* = 2.86). A demographic screener survey was used to determine participant eligibility for both groups (see below). All participants were either recruited from introductory psychology classes and compensated with course credit or recruited from the general campus community and paid $18 for their participation. All procedure was approved by the university’s institutional review board and conducted in accordance with the American Psychological Association’s ethical standards.

### Eligibility Criteria

We relied on several pieces of culturally relevant information, including behavioral information such as language preferences, to go beyond racial or ethnic self-identification to characterize our groups based on criteria employed in previous studies of culture and emotion [see Soto et al. (2005) and Soto and Levenson (2009) for full discussion of the rationale behind the criteria]. European Americans must have been born and raised in the United States and had to self-identify as White or European American. Participants also had to report that their parents and grandparents were born in the United States and identified as White or European American. In addition, European American participants had to report being of Christian or Catholic religion, or growing up with these religions being practiced in their households. Finally, participants had to report that over 50% of their friends while growing up and over 40% of their neighborhood while growing up were of European American background.

Asian American participants had to self-report their ethnicity as Asian or East Asian (e.g., Chinese, Korean, Japanese, and Vietnamese) and have been born either in an East Asian country or in the United States. South Asian participants from countries such as India, Pakistan, or Bangladesh were not eligible. In addition, participants’ parents and grandparents also had to meet the same birth-country requirements. Furthermore, participants had to be conversant, though not fluent, in both English and in the Asian language of their culture of origin. There were no religious criteria for the Asian American participants. The criteria around childhood friends and neighborhood were also not applied to this group. While the original criteria were developed for participants living in a large metropolitan area where exposure to culturally similar others is common, this assumption would have been an unrealistic standard for the East Asian and Asian American participants in the community from which participants in the current study were sampled (University Park, PA, United States).

### Procedure

Data used for the present study were collected as part of a large multi-method project investigating cultural differences in the experience and regulation of physiological, behavioral, and self-reported responses to emotional stimuli. Upon arriving at the lab room, participants signed the informed consent form and sat in a comfortable chair 3 feet away from a 19″ LCD monitor. Participants completed a series of questionnaires including measures of emotion, depression, life satisfaction, collective self-esteem, the importance of their racial group membership to their identity (see below), and other measures outside of the scope of the present study. After this point, an experimenter of the same gender applied the physiological sensors to participants. Participants then watched a total of five film clips previously used in emotion regulation research (Gross and Levenson, 1993; Kunzmann et al., 2005) while their facial and physiological responses were collected. After each film, participants completed a self-report measure of emotion. All films were between 52 and 62 s in duration, with the exception of the first film, which lasted 22 s. Film 1 was the same across all participants and was a neutral film (seagulls flying over a beach). Films 2–4 were disgust films. The first disgust film (Film 2) always depicted an eye operation and was not associated with any specific emotion regulation instructions. The next two films were of a burn victim’s skin graft and an arm amputation, and participants were asked to either amplify or suppress their emotional expression while viewing the films. The order of regulation instructions and the actual film presentation for films 3 and 4 were counterbalanced. Film 5 was a slightly positive film (nature scenes) used to help participants recover from negative emotions induced by previous films [see Soto et al. (2016) for more detailed information about the methods and procedures].

The fact that this convenience dataset consisted only of neutral, relaxing, and disgust elicitors limited the scope of our emotional fit variable. However, given that disgust reactivity does not tend to vary greatly across cultures (Rozin et al., 2008), we also thought this would provide a more conservative test of our research question pertaining to cultural moderation. In addition, examining emotional fit in response to neutral stimuli may provide important information that has been hitherto unexamined given that neutral stimuli are often processed similarly as negative stimuli (Codispoti et al., 2001; Lee et al., 2008), especially so among clinical populations (Felmingham et al., 2003; Leppänen et al., 2004). Thus, responses to the neutral stimuli could reflect individual differences in responding that could lead to variability in emotional fit that may be meaningfully related to well-being outcomes.

The present study examined emotional fit at the first three time points prior to the introduction of emotional regulation instructions – emotional fit at baseline (Time 0), emotional fit in response to neutral film (Time 1), and emotional fit in response to the disgust film (Time 2). We did not include time points after emotion regulation instructions were presented because the impact of these instructions on emotional fit is outside of the scope of the present study. Because the collection of behavioral and physiological data began with the introduction of neutral film, baseline response (Time 0) consisted of the self-report measure of emotion only. Responses to neutral film (Time 1) and disgust film (Time 2) consisted of self-report, behavioral, and physiological responses.

### Measures

#### Satisfaction With Life Scale

Participants completed a five-item measure of life satisfaction. The SWLS assesses global judgments of satisfaction with one’s life (SWLS; Diener et al., 1985). Participants are asked to rate their responses to questions such as “in most ways my life is close to my ideal” and “the conditions of my life are excellent,” using a 7-point Likert scale (1 = strongly disagree to 7 = strongly agree). Higher scores indicate greater satisfaction with life. The SWLS has shown good internal consistency in previous studies, with alpha coefficients ranging from 0.79 to 0.89 (Pavot and Diener, 1993). Cronbach’s alpha coefficients in the current sample were 0.79 for Asian Americans and 0.84 for European Americans, indicating acceptable to good reliability.

#### Center for Epidemiologic Studies Depression Scale

The CES-D is a 20-item self-report inventory of depressive symptoms (CES-D; Radloff, 1977). Participants use a 4-point Likert scale (0 = rarely or none of the time to 3 = most or all of the time) to rate the degree to which they experienced, over the past week, major symptoms of depression including depressed mood, feelings of guilt and worthlessness, feelings of helplessness and hopelessness, psychomotor retardation, loss of appetite, and sleep disturbance. Higher scores indicate greater depressive symptoms. The CES-D has shown good internal consistency with alpha coefficients ranging from 0.85 to 0.90 in previous studies (Radloff, 1977). In the current study, the CES-D also indicated good internal consistency with an alpha coefficient of 0.85 for both Asian Americans and European Americans.

#### Collective Self-Esteem Scale – Private Collective Self-Esteem and Importance to Identity Subscales

The 4-item private collective self-esteem and 4-item importance to identity subscales of the CSES were used to measure participants’ positive collective identity and identification with their group (CSES; Luhtanen and Crocker, 1992). The private collective self-esteem refers to one’s evaluation of how good one’s ethnic group is. Importance to identity (henceforth, identity) assesses how important one’s ethnic group is to one’s self concept. The public collective self-esteem (one’s perception of how others evaluate one’s ethnic group) and membership esteem (one’s perception of how good of a member one is for one’s ethnic group) subscales were not included because they were less relevant to the focus of the present study. Participants use a 7-point Likert Scale (1 = strongly disagree to 7 = strongly agree) to rate their collective self-esteem. Higher scores indicate greater collective self-esteem. The original validation study (Luhtanen and Crocker, 1992) reported alpha coefficients ranging from 0.73 to 0.85, indicating acceptable to good internal consistency. In the current sample, the private collective self-esteem subscale indicated acceptable internal consistency with alpha coefficients of 0.79 for Asian Americans and 0.72 for European Americans. The alpha coefficients for the identity subscale were 0.79 and 0.86 for Asian Americans and European Americans, respectively, indicating acceptable to good internal consistency.

#### Multidimensional Inventory of Black Identity – Centrality Subscale

To assess the degree to which participants identify with their ethnic group (referred to as racial centrality hereafter), we used the 8-item centrality subscale of the MIBI (MIBI; Sellers et al., 1997). The centrality subscale of the MIBI assesses a broader concept of group identification than the CSES identity subscale. In addition to assessing the degree to which ethnic group membership is central to one’s core self-concept, the MIBI centrality scale also captures participants’ sense of connection/belonging to other members of their ethnic group. Because the items in the original MIBI were developed for African Americans only, we modified the wording of items to accommodate other ethnic groups as well. Items include “overall, being of my racial group has very little to do with how I feel about myself” and “I have a strong sense of belonging to people of my racial group.” This modification has been used previously with ethnic minority groups other than African Americans (Perez and Soto, 2011). Participants rated their response using a 7-point Likert scale (1 = strongly disagree to 7 = strongly agree), and higher score indicated greater importance of racial group membership to their identity. The internal consistency of the centrality subscale of the MIBI was 0.77 in the original validation study, which indicates acceptable consistency (Sellers et al., 1997). The current sample also indicated acceptable consistency, with alpha coefficients of 0.79 and 0.77 for Asian Americans and European Americans, respectively.

#### Self-Reported Emotional Experience

At six different time points throughout the experiment (i.e., at the beginning of the experiment, and after each of five films), participants were asked to use a 9-point Likert scale (0 = none and 8 = the most in my life) to rate their current experience of 16 different emotions: interest, happiness, surprise, amusement, contentment, relief, anxiety, sadness, annoyance, disgust, embarrassment, boredom, fear, anger, contempt, and stress. This rating scale has been used to measure the experience of specific emotions in previous emotion research (Ekman et al., 1980; Soto et al., 2005).

#### Facial Emotional Expression

Participants’ facial expressions during the presentation of films were video recorded and then coded into six discrete emotions (happiness, sadness, anger, surprise, fear, and disgust) using the commercial face reading software FaceReader v. 6.1 (Noldus, 2014). FaceReader objectively estimates the presence of emotion expressions by utilizing over 500 facial landmark cues typically present in emotion expressions as well as specific action units as defined by Paul Ekman’s facial affect coding system. For each video frame (image) FaceReader supplies a “confidence score” between 0 and 1 representing the likelihood that each discrete emotion is present. FaceReader was trained on over 10,000 expert-coded images and has demonstrated high accuracy for emotion expression classification (Lewinski et al., 2014).

For the present study, we averaged confidence estimates for the presence of each emotion expression over the 1-min film presentation period. This resulted in six scores per film clip per participant representing the average likelihood that each of the emotions were present over the film’s presentation.

#### Physiological Response

Electrocardiography (EKG) and skin conductance level (SCL) were recorded using a Mindware impedance cardiograph (MW2000) in conjunction with the Biopac MP150© device consisting of an eight-channel polygraph and a microcomputer. All physiological data were collected second-by-second using AcqKnowledge© software. EKG, which provides a measure of cardiac activity, was measured through three Biopac pre-gelled, self-adhering, disposable electrodes placed at three places on the torso: the right clavicle at the midclavicular line, just above the last bone of the ribcage at the left midaxillary line, and just below the last bone of the ribcage at the right midaxillary line. Cardiac impedance was collected with four self-adhering electrodes – one placed at the suprasternal notch (jugular notch), one at the inferior end of the sternum (xiphoid process), and two on the back (one located roughly at the fourth cervical vertebra and one located roughly at the eighth thoracic vertebrae). MindWare Impedance Cardiography and MindWare HRV 2.51 software (MindWare Technologies Ltd., Gahanna, OH, United States) were used to clean raw data and extract the systolic time intervals (PEP, LVET) and heart rate variability (RSA) using spectral analysis. Clear artifacts in EKG data were deleted and excluded from analyses. In addition, SCL was measured using two disposable electrodes filled with isotonic recording gel that were placed on the middle phalange of the second and fourth fingers of the non-dominant hand. While indicators of both sympathetic (SNS) and parasympathetic nervous system (PNS) arousal can be obtained from analysis of physiological data, the present study focused on the pattern of SNS arousal. SNS indices include HR, cardiac output (CO), stroke volume (SV), left ventricular ejection time (LVET), cardiac impedance (Zo), pre-ejection period (PEP), and SCL. HR is the number of contractions of the heart per minute. CO is a measure of the overall volume of blood being pumped by the heart per minute. SV represents the volume of blood ejected by the left ventricle of the heart in one beat. LVET is a measure of myocardial contractility. Zo is an indicator of blood flow through the thoracic cavity. PEP is an indicator of sympathetic myocardial drive and indicates the interval between onset of the EKG Q-wave and onset of the left ventricular ejection. SCL is an index of sweat gland activity at the surface of the skin.

#### Emotional Fit Indices

Following a calculation method used in previous studies of emotional fit with culture (De Leersnyder et al., 2014, 2015), three types of emotional fit with individuals’ own culture (i.e., Asian American and European American) were calculated using self-report emotion ratings (self-report emotional fit), behavioral responses (behavioral emotional fit), and physiological responses (physiological emotional fit). The means and variances of all variables used to calculate emotional fit are presented in **Table 1**.

**Table 1:** Means and variances of self-report emotion, facial expression, and physiological response variables used to calculate emotional fit.

	Time 0: Baseline			Time 1: Neutral film			Time 2: Disgust film		
	*n*	Mean	Variance	*n*	Mean	Variance	*n*	Mean	Variance
**Self-reports**									
Interest	127	5.70	2.39	127	4.21	4.95	127	3.91	4.63
Happiness	127	5.20	2.14	127	4.54	3.57	127	3.57	4.04
Surprise	127	3.16	4.71	127	2.59	4.45	127	3.61	6.48
Amusement	127	4.54	3.08	127	3.19	3.92	127	2.68	4.30
Contentment	127	5.21	2.06	127	4.24	3.66	127	3.27	3.86
Relief	127	3.90	2.84	127	3.02	4.49	127	2.33	4.22
Anxiety	127	2.87	3.52	127	2.06	3.61	127	2.19	4.50
Sadness	127	1.28	2.36	127	0.93	2.18	127	1.31	2.90
Annoyance	127	1.23	2.02	127	1.48	3.39	127	1.87	4.72
Disgust	127	0.65	1.33	127	0.43	1.04	127	2.62	7.44
Embarrassment	127	0.91	1.56	127	0.55	1.01	127	0.69	1.90
Boredom	127	2.25	3.70	127	2.86	5.30	127	2.61	5.32
Fear	127	0.98	2.05	127	0.72	2.03	127	1.37	3.73
Anger	127	0.60	1.27	127	0.50	1.36	127	0.76	2.39
Contempt	127	1.47	3.98	127	1.09	2.91	127	1.13	2.60
Stress	127	2.73	4.91	127	1.80	3.12	127	2.46	4.38
**Facial expressions**									
Happiness	–	–	–	122	0.03	0.01	120	0.05	0.02
Sadness	–	–	–	122	0.11	0.05	120	0.09	0.04
Anger	–	–	–	122	0.10	0.04	120	0.16	0.06
Surprise	–	–	–	122	0.10	0.05	120	0.07	0.03
Fear	–	–	–	122	0.02	0.01	120	0.02	0.01
Disgust	–	–	–	122	0.08	0.03	120	0.17	0.05
**Physiological responses**									
HR	–	–	–	90	0.45	0.02	93	0.38	0.03
CO	–	–	–	89	0.74	0.02	92	0.37	0.01
SV	–	–	–	89	0.73	0.01	92	0.39	0.01
LVET	–	–	–	90	0.81	0.03	93	0.40	0.01
Zo	–	–	–	95	0.56	0.01	95	0.93	0.01
PEP	–	–	–	90	0.77	0.01	93	0.65	0.01
SCL	–	–	–	92	0.60	0.04	92	0.71	0.02

*Physiological responses were standardized. Zo and PEP were reversed coded so that the greater score indicates greater sympathetic activations for all physiological variables.*

In order to calculate self-report emotional fit, we first calculated the group’s average rating for each of the 16 different emotions excluding the respondents’ own scores, which constituted the group’s average emotional profile. We then correlated each individual’s emotional profile consisting of 16 emotions to the group’s average emotional profile. The derived correlation coefficients were Fisher’s *z*-transformed in order to achieve a normal distribution of data. The final correlation coefficient for each individual served as self-report emotional fit score – the degree to which individual’s emotional profile resembles the normative emotional profile of one’s group. This process was repeated three times for each of the three time points (baseline, Films 1 and 2), resulting in three separate self-report emotional fit scores for Times 0, 1, and 2.

Behavioral emotional fit was calculated using the facial expression data. Six emotions used for behavioral emotional fit were happiness, sadness, anger, surprise, fear, and disgust. Following the same procedure as self-report emotional fit, the group’s average behavioral emotional profile was derived from the group’s average score on each of the six different emotions excluding the respondents’ own scores. Then the group’s emotional profile was correlated to each individual’s emotional profile, and the Fisher’s *z*-transformation was applied. This process was repeated two times, each using the responses to Films 1 and 2, resulting in two separate behavioral emotional fit scores for each individual in Times 1 and 2.

For calculating physiological emotional fit, we used seven different indices of sympathetic activation collected during the first two films. These were HR, CO, SV, LVET, Zo, PEP, and SCL. Among these, Zo and PEP decreases as SNS activity increases. Thus, Zo and PEP indices were reverse coded by multiplying them by −1, so that the increase in number would indicate greater SNS arousal. In addition, each of these indices were originally on different scales. Therefore, we standardized the scores using the formula: (*x-x*_min_)/(*x*_max_-*x*_min_), which transformed the data into a 0–1 scale. The rest of the process of calculating emotional fit was identical to that of self-report and behavioral emotional fit. We first calculated the group’s average scores for each of the seven sympathetic indices while excluding the respondents’ own score and used it as the group’s average emotional profile. This was correlated to individual’s profile of physiological responses. The correlation coefficients were then Fisher’s *z*-transformed. The process was repeated two times for each individual using the responses to Films 1 and 2, which resulted in two separate physiological emotional fit scores for each individual in Times 1 and 2.

## Results

### Data-Analytic Approach

To test the link between participants’ well-being and emotional fit and whether culture moderates this link, we conducted a series of multiple regression analyses. In these analyses, Emotional Fit variables were always entered as Step 1, followed by Culture in Step 2, and the interaction between Emotional Fit and Culture in Step 3 to test for the hypothesized moderation of culture on the link between emotional fit and well-being. When significant interactions between emotional fit and culture emerged, the identified interaction effects were decomposed using a simple slopes analysis (Aiken et al., 1991). In addition, based on prior evidence suggesting gender differences in response to disgust (e.g., Schienle et al., 2005; Rohrmann et al., 2008), we examined the effects of gender on (a) the emotional responses to the disgust film and (b) our indices of emotional fit. Some gender differences emerged across specific facial expressions in response to disgust, and behavioral emotional fit also varied significantly by gender^1^ As a result, we re-ran our regression models controlling for gender, and this did not change any of our reported findings. Therefore, we report the models without gender for the sake of parsimony.

In reporting of the results, we focus on the main effect of emotional fit in Step 1 and interaction between emotional fit and culture in Step 3. Correlations between emotional fit and well-being variables and descriptive statistics are presented in **Table 2**. For our primary analyses (self-report emotional fit at time 0), we chose not to correct the alpha level (0.05) to preserve power and because we were testing a priori hypotheses (confirmatory analyses) and only conducted five regressions to test two questions (Rothman, 1990; Proschan and Waclawiw, 2000; van Belle, 2008; Rubin, 2017). For the exploratory analyses, we employed the Bonferroni correction given the large number of tests conducted. In all, we tested how three types of fit (self-report, behavioral, and physiological) relate to two types of outcomes (individual well-being and collective aspects of well-being) using a total of 30 regressions relating to variations in the specific outcome variables and time points considered. Thus, the adjusted *p*-value of 0.002 (0.05/30) was used to re-evaluate any of the significant findings that emerged from analysis using an uncorrected *p*-value. We chose to present the results of the test *both* before and after the Bonferroni correction given the recommendation that corrections for multiple comparisons also has the drawback of reducing power (Rothman, 1990).

**Table 2:** **(A)** Correlations between emotional fit and well-being in Asian Americans (below the diagonal) and in European Americans (above the diagonal), and **(B)** mean and standard deviation of emotional fit and well-being variables in Asian Americans and European Americans.

(A)												
	**1**	**2**	**3**	**4**	**5**	**6**	**7**	**8**	**9**	**10**	**11**	**12**
(1) EF_T0–SR_	1	0.65^∗∗^	0.66^∗∗^	0.18	0.12	−0.04	−0.01	−0.28^∗^	0.21	0.03	0.04	0.01
(2) EF_T1–SR_	0.63^∗∗^	1	0.83^∗∗^	0.15	0.21	−0.09	−0.03	−0.21	0.05	0.00	0.02	0.13
(3) EF_T2–SR_	0.38^∗∗^	0.48^∗∗^	1	0.19	0.12	−0.09	0.01	−0.20	0.13	−0.03	0.02	0.12
(4) EF_T1–PHY_	−0.10	0.07	0.07	1	−0.06	−0.06	−0.14	−0.18	0.14	−0.11	−0.06	0.14
(5) EF_T2–PHY_	−0.03	0.08	−0.03	0.51^∗∗^	1	−0.08	0.04	0.01	0.09	−0.06	0.02	−0.04
(6) EF_T1–BEH_	0.12	0.12	0.06	−0.36^∗^	−0.32	1	0.58^∗∗^	−0.02	0.02	−0.07	−0.11	−0.10
(7) EF_T2–BEH_	−0.07	0.01	0.01	0.14	0.05	0.36^∗^	1	−0.09	0.10	−0.06	0.01	−0.05
(8) Depression	−0.39^∗∗^	−0.37^∗∗^	−0.19	0.02	−0.12	−0.26	−0.12	1	−0.40^∗∗^	−0.04	−0.10	−0.09
(9) Life satisfaction	0.35^∗^	0.40^∗∗^	0.26	0.21	0.01	0.04	0.13	−0.55^∗∗^	1	0.16	0.05	0.01
(10) Private collective self-esteem	0.39^∗∗^	0.10	0.22	0.04	0.04	0.07	−0.05	−0.41^∗∗^	0.20	1	0.24^∗^	0.23^∗^
(11) CSES identity	0.19	0.05	0.29^∗^	0.01	0.14	0.04	0.05	−0.22	0.13	0.43^∗∗^	1	0.69^∗∗^
(12) Racial centrality	0.31^∗^	0.12	0.11	0.21	0.33^∗^	0.13	0.07	−0.24	0.12	0.44^∗∗^	0.71^∗∗^	1

*^∗^p < 0.05; ^∗∗^p < 0.01.*												

(B)			
	Mean (*SD*)		
	All samples	Asian Americans	European Americans
(1) EF_T0–SR_	1.16 (0.45)	1.17 (0.49)	1.15 (0.42)
(2) EF_T1–SR_	0.91 (0.48)	0.87 (0.51)	0.94 (0.46)
(3) EF_T2–SR_	0.63 (0.40)	0.58 (0.38)	0.67 (0.42)
(4) EF_T1–PHY_	1.62 (1.94)	1.33 (0.67)	1.84 (2.48)
(5) EF_T2–PHY_	1.94 (0.81)	1.93 (0.70)	1.94 (0.89)
(6) EF_T1–BEH_	0.31 (0.45)	0.21 (0.38)^∗^	0.37 (0.48)^∗^
(7) EF_T2–BEH_	0.61 (0.72)	0.64 (0.81)	0.59 (0.65)
(8) Depression	31.98 (7.39)	31.85 (7.44)	32.08 (7.41)
(9) Life satisfaction	23.76 (5.61)	22.50 (5.53)^∗^	24.63 (5.54)^∗^
(10) Private collective self-esteem	23.65 (3.47)	23.75 (3.83)	23.59 (3.22)
(11) CSES identity	15.32 (6.24)	19.58 (4.51)^∗∗∗^	12.37 (5.55)^∗∗∗^
(12) Racial centrality	29.56 (9.65)	36.46 (7.75)^∗∗∗^	24.77 (7.78)^∗∗∗^

*Means with asterisks significantly differ between groups. ^∗^p < 0.05; ^∗∗∗^p < 0.001.*

### Self-Report Emotional Fit

We first examined the link between self-report emotional fit at Time 0 (EF_T0-SR_) and individual well-being variables, and whether culture moderated this relationship. There was a significant main effect of EF_T0-SR_ on depression, with higher emotional fit predicting reduced depression, β = −5.45, *t*(1, 125) = −3.91, *p* < 0.001. As predicted, the interaction between EF_T0-SR_ and culture on depression was not significant. Similarly, a significant main effect of EF_T0-SR_ was found in predicting life satisfaction, such that higher emotional fit predicted greater life satisfaction, β = 3.29, *t*(1, 125) = 3.05, *p* = 0.003. As hypothesized, culture did not moderate this relationship either. Next, we tested the link between self-report emotional fit at the remaining time points and individual well-being variables. The results were largely consistent with Time 0 findings. There was a significant main effect of self-report emotional fit at Time 1 (EF_T1-SR_) on depression, such that higher emotional fit predicted reduced depression, β = −4.26, *t*(1, 125) = −3.21, *p* = 0.002. There was a significant main effect of EF_T1-SR_ on life satisfaction with higher emotional fit predicting greater life satisfaction, β = 2.50, *t*(1, 125) = 2.44, *p* = 0.016. The same pattern of results emerged with self-report emotional fit at Time 2 (EF_T2-SR_). There were significant main effects of EF_T2-SR_ on both depression and life satisfaction, β = −3.56, *t*(1, 124) = −2.19, *p* = 0.03, β = 2.75, *t*(1, 124) = 2.24, *p* = 0.027, respectively. After applying a Bonferroni correction to these exploratory analyses at Times 1 and 2, only the relationship between EF_T1-SR_ and depression remained significant. Culture did not moderate any of the associations between self-report emotional fit at T1 and T2 and individual well-being.

**FIGURE 1:**
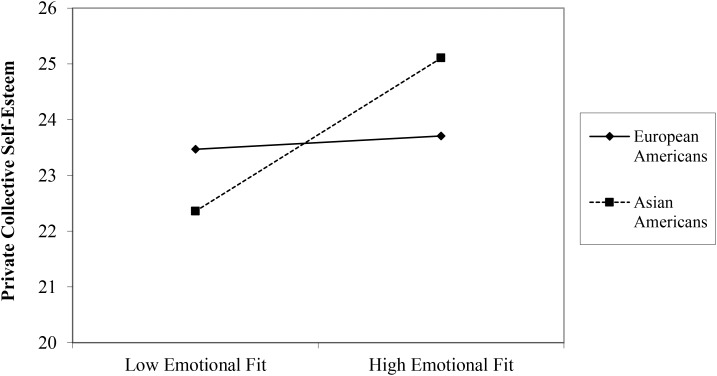
The relationship between self-report emotional fit at Time 0 and private collective self-esteem for European Americans and Asian Americans.

Next, looking at the effects of emotional fit on collective aspects of well-being, there was a significant main effect of emotional fit at Time 0 on collective self-esteem (i.e., one’s evaluation of how good one’s ethnic group is) with higher emotional fit predicting greater collective self-esteem, β = 1.64, *t*(1, 125) = 2.43, *p* = 0.017. As hypothesized, this main effect was qualified by a significant interaction between EF_T0-SR_ and culture, β = 2.79, *t*(3, 123) = 2.08, *p* = 0.04. A follow-up simple slopes analysis revealed that the simple slope of the regression of collective self-esteem onto EF_T0-SR_ for Asian Americans was significant (simple slope = 3.05), *t*(123) = 3.20, *p* = 0.002, with higher EF_T0-SR_ predicting greater collective self-esteem (**Figure 1**). In European Americans, the relationship between collective self-esteem and EF_T0-SR_ was non-significant (simple slope = 0.27), *t*(123) = 0.28, *p* = 0.779. These findings were specific to Time 0 Emotional Fit. There were no significant main effects of EF_T1-SR_ and EF_T2-SR_ on collective self-esteem, and no cultural moderation was found at these additional time points. The effects of emotional fit on measures of how important one’s ethnicity is to one’s own self-concept (CSES identity and racial centrality) were non-significant across all three time points. That is, EF_SR_ in Times 1, 2, and 3 did not predict either CSES identity or racial centrality, and there was no cultural moderation, all *ps* > 0.05.

### Additional Indices of Emotional Fit

**FIGURE 2:**
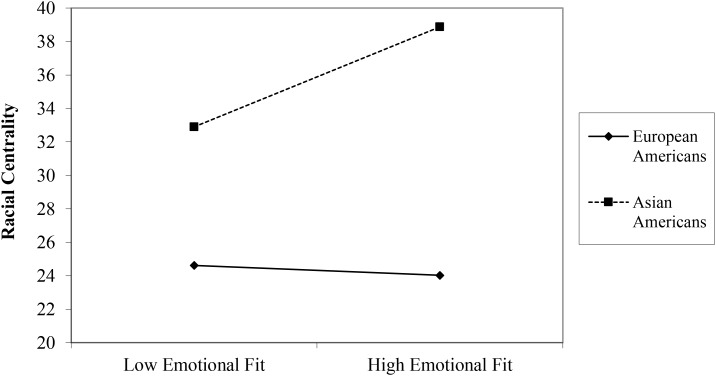
The relationship between physiological emotional fit at Time 2 and racial centrality for European Americans and Asian Americans.

Next, we explored whether behavioral and physiological indices of emotional fit predicted individual and collective aspects of well-being. Both behavioral emotional fit at Time 1 (EF_T1-BEH_) and Time 2 (EF_T2-BEH_) did not predict any of the outcome variables, and there was no interaction between EF_BEH_ and culture. Looking at physiological indices of emotional fit, there was no main effect of physiological emotional fit at Time 1 (EF_T1-PHY_) on any of the outcome variables, and no cultural moderation was found. Similarly, there was no main effect of physiological emotional fit at Time 2 (EF_T2-PHY_) on any of the outcome variables. However, there was a marginally significant interaction effect between EF_T2-PHY_ and culture in predicting racial centrality, β = 4.03, *t*(3, 91) = 1.92, *p* = 0.058. A follow-up simple slopes analysis indicated that the simple slope of the regression of racial centrality onto EF_T2-PHY_ for Asian Americans was significant (simple slope = 3.67), *t*(91) = 2.09, *p* = 0.04, with higher EF_T2-PHY_ predicting greater racial centrality (**Figure 2**). In contrast, the simple slope was non-significant in European Americans (simple slope = −0.36), *t*(91) = −0.32, *p* = 0.753. This marginally significant interaction became non-significant when the Bonferroni corrected *p*-value was applied.

## Discussion

The present study examined the association between emotional fit and individual and collective aspects of well-being and the role of culture in this relationship. Emotional fit based on self-report ratings of emotions significantly predicted individual well-being including reduced depression and greater life satisfaction in both Asian Americans and European Americans. In contrast, self-report emotional fit in the absence of laboratory stimuli predicted collective aspects of well-being, particularly collective self-esteem only in Asian Americans. In addition, emotional fit based on physiological response to a strong negative stimulus predicted greater identification with one’s group only in Asian Americans, though this cultural moderation was only marginally significant in the initial test and disappeared when the Bonferroni correction was applied.

### Self-Report Emotional Fit

Emotional fit based on self-reported emotions in all three time-points was associated with individual well-being (i.e., lower depression and greater life satisfaction) across cultures. This finding is in line with the view that while there may be different cultural mandates for well-being in interdependent and independent cultures (e.g., social harmony in Japan and personal control in United States; Kitayama et al., 2010), being in alignment with one’s own cultural norms around emotion is generally important for individual well-being across cultures. It has been shown that even though different emotions are preferred in Japan and the United States, the experience of culturally preferred emotions was associated with happiness in both cultures (Kitayama et al., 2006). In a similar vein, experiencing a culturally normative pattern of emotions has been found to be important for psychological well-being in both independent and interdependent cultures, although the specific contexts in which emotional fit becomes crucial varies depending on respective cultural values (De Leersnyder et al., 2015). Because people’s emotions are shaped by how they perceive and appraise their environment (Ellsworth and Scherer, 2003), their fit with the average emotional pattern of others in the same culture may represent their level of sharing and participating in the predominant world-view of that culture. Thus, emotional fit to a certain extent may reflect a general level of social adjustment (De Leersnyder et al., 2011), which may have universal implications for one’s psychological well-being.

While we have conceptualized the above relationship as one where emotional fit with one’s group might lead to increased well-being, we can also consider the pathway in which individual well-being leads to increased emotional fit. For instance, the cultural norms hypothesis of depression (Chentsova-Dutton et al., 2007) suggests that the symptoms of depression (i.e., impaired concentration, low energy, and anhedonia) may impair individuals’ abilities to attend to and enact cultural norms and ideals regarding emotion and emotional expression. Indeed, it has been demonstrated that depressed individuals showed lower emotional fit with their cultural group than did non-depressed individuals (Chentsova-Dutton et al., 2007). These findings demonstrate that perhaps individuals who have lower well-being and greater depression may have more difficulty responding in a culturally concordant manner. As such, more research is needed in order to establish the directionality of the relationship between emotional fit and well-being.

In contrast to the individual well-being findings, culture moderated the relationship between self-report emotional fit and collective identity, particularly, individuals’ evaluation of their own cultural group (collective self-esteem). In Asian Americans, greater emotional fit predicted more positive evaluation of their own cultural group, whereas such a relationship was not present in European Americans. People generally experience similarity as safe and comforting, and similarity leads to greater liking (Montoya et al., 2008). This may be especially so in cultures where social harmony and conformity are greatly valued and practiced. Previous research has shown that people in collectivistic societies conform more than those in individualistic societies (Bond and Smith, 1996). It is possible that this greater importance of similarity in East Asian cultures leads to greater liking or more positive evaluation of the group that one also shares an emotional response pattern with. Alternatively, individuals may be more motivated to behave consistently with the group when they feel positively about their own cultural group. It is possible that we see this pattern only in Asian American individuals because conformity, in general, is practiced more in collectivistic than individualistic societies (Bond and Smith, 1996).

On the other hand, the inconsistency between one’s own emotions and the modal emotional pattern of one’s culture may be more self-threatening in interdependent culture. Negative evaluation of a group that is seen as dissimilar to oneself may represent an attempt to reconcile this threat to self by degrading dissimilar others and in turn preserving or enhancing the self. Alternatively, however, the experience of dissimilarity may lead to negative evaluation of both the individual and group in interdependent cultures. Extensive research on interdependent self-construal in interdependent cultures (e.g., Markus and Kitayama, 1991) suggests that there may be a greater overlap between individual and collective selves in Asian cultures. Although the evaluation of individual self (e.g., personal self-esteem) was not measured in the current study, it is possible that reduced fit with other Asian Americans led to more negative evaluations of the individual self, which in turn spilled over to the evaluation of their collective self.

In addition to the possible role of interdependence and collectivist values in the present findings, the role of Asian Americans’ position as a racial minority group in the United States cannot be ignored. For instance, the status of a racial minority and the repeated experience of being marginalized may have led Asian Americans to seek belonging and to place a greater value on the group through which they can fulfill such a need. As such, Asian Americans who share emotional similarity to the members of their cultural group may be able to more readily satiate their need for belonging through their group membership, and in turn, evaluate their group more positively. Additionally, because a minority often experiences being perceived as representing one’s broader minority group as a whole, Asian Americans may be more aware of and sensitive to how their individual behavior reflects on outside perceptions of their group as a whole. In the presence of this heightened sense of prescribed connection between their own behaviors and the outside perception of their group, Asian Americans may experience the group with which they share emotional similarity (i.e., greater emotional fit) less effortful to represent, and thus, leading to greater liking or more positive evaluation.

Interestingly, the results relating to self-report emotional fit and collective self-esteem were specific to emotional fit at baseline before any specific laboratory stimuli were presented. This could be because reflective responses to a strong emotional stimulus may override individual or cultural variability in emotional patterns, leading to too little variability in emotional fit indices, which in turn may limit the possibility of identifying any meaningful patterns between emotional fit and outcome measures. In fact, the variance in self-report emotional fit was lowest in Time 2 when the fit was measured in response to a strong negative stimulus. The pattern of results regarding individual well-being is somewhat consistent with this point as well. While the effect of self-report emotional fit on individual well-being was observed at all three time points, the magnitude of effect decreased from emotional fit at Time 0, to Time 1 (in response to neutral film), and to Time 2 (in response to disgust film), and some of the Times 1 and 2 effects were eliminated when employing the Bonferroni correction.

### Additional Indices of Emotional Fit

Another aim of this study was to explore whether any of the effects found with self-report emotional fit is replicated with other indices of emotional fit such as behavioral and physiological emotional fit. We did not find the comparable patterns of results with other indices of emotional fit, which is consistent with the dual-process perspective suggesting that there is little response coherence between reflective and automatic emotion systems (Evers et al., 2014). In addition, indices of emotional fit at different levels were largely uncorrelated to each other, although emotional fit indices within the same level (e.g., self-report, physiology) were generally related to each other.

Behavioral emotional fit in response to both neutral and disgust films did not predict any individual and collective aspects of well-being. Similarly, physiological emotional fit in response to the neutral film did not predict any of the outcome variables. However, a marginally significant interaction pointed to a pattern consistent with our prediction such that higher physiological emotional fit in response to disgust film was associated with greater racial centrality in Asian Americans, whereas there was no such relationship in European Americans. In other words, the perceived level of group identification (racial centrality) was mirrored in greater individual-group synchrony in automatic responses to a strong emotional situation in Asian Americans. It is conceivable that when members of interdependent culture identify with their group, their collective identity gets deeply internalized to the point that this is reflected in a greater physiological concordance with their group members. This result, however, became non-significant after employing the Bonferroni correction. Given the small sample size, we believe this finding may nevertheless be worth testing in future studies, especially since we observed the similar pattern found in the primary analyses (emotional fit relating to collective aspects of well-being for Asian Americans only), although only in response to a strong negative stimulus (Time 2). Future studies aiming to measure physiological emotional fit may note that in the absence of a stimulus to respond (no stimuli or neutral stimuli) there may be too much variability/physiological noise across subjects to be able to calculate a meaningful fit index. However, the introduction of a punctate stimulus may organize the physiological system enough to be able to calculate the fit indices discussed. The variance in physiological emotional fit in Time 1 was considerably greater than that of Time 2, which further support this possibility. Thus, while these findings are not robust they are suggestive of a possible future direction to pursue when there is adequate power to test the hypothesis.

### Limitations and Future Directions

The current study has a few important limitations that are worth noting. First, while we used data from previous study that allowed us to also explore behavioral and physiological emotional fit in addition to self-report emotional fit, we did not have behavioral and physiological emotional fit indices at Time 0. Thus, we cannot know whether our self-report emotional fit findings from Time 0 will be corroborated with behavioral and physiological emotional fit measured in the same context. In addition, the choice of emotion elicitors was restricted by the nature of convenience dataset. In particular, given that disgust may be an emotion with the least cultural variability, the use of the disgust film at Time 2 allowed for a more conservative test of our research question but also may have underestimated the impact of emotional fit. Future studies employing varying indices of emotional fit across diverse emotional contexts are needed for a more in-depth investigation into the effects of emotional fit. Second, our study is cross-sectional, and thus cannot answer questions regarding the directionality in the observed links between emotional fit and well-being. Additionally, the design of the current study does not allow us to explore the specific mechanisms underlying the relationship between emotional fit and well-being as well as the cultural moderation observed in predicting collective aspects of well-being. Important next steps would be to examine the causality in the link between emotional fit and well-being through a longitudinal design or a laboratory experiment where emotional fit is manipulated (e.g., Livingstone et al., 2011) and through what processes such causal effects emerge. Third, it will be important to replicate these results in East Asians residing in East Asian countries to disentangle the potential role of interdependence with that of being a minority experience in the current finding. Fourth, careful studies examining gender effects on emotional fit would also be a fruitful avenue of future research. Based on the observed gender differences in behavioral emotional fit, it may be worth examining gender-specific emotional fit (emotional fit calculated using a same-gender reference group) and how it relates to well-being. Lastly, prior studies examining emotional fit using the same profile correlation approach have used a relatively larger sample (e.g., *N* = 266 in Study 3 in De Leersnyder et al., 2015) compared to the current study. The relatively small size of the current sample, especially in regard to exploratory analyses with physiological emotional fit (Asian American *n* = 39, European American *n* = 56) may have limited our ability to detect significant relationships between primary variables of interest. Although this preliminary result is interesting, future studies using a larger sample should further examine this finding to draw more meaningful conclusions.

## Conclusion

Individuals must constantly navigate through their social worlds while paying simultaneous attention to both their individual needs and behaviors and the needs and behaviors of those around them. However, the extent to which individual and group behaviors *fit* with each other can vary meaningfully across cultural groups as can the relationship between this fit and well-being. The present study revealed that emotional fit based on individuals’ subjective emotional experience predicted individual well-being across cultures, but predicted collective self-esteem only in Asian Americans. Being the first study to examine the relationship between emotional fit and collective aspects of well-being, the current finding adds to the growing research attempting to understand emotions as social and interpersonal processes that are naturally imbedded in cultural contexts. We believe this underscores the need to consider, not only how emotions may conform to normative patterns in one’s cultural milieu, but that this degree of fit may impact members of different cultures in different ways.

## Ethics Statement

This study was carried out in accordance with the recommendations of the American Psychological Association’s ethical standards with written informed consent from all subjects. All subjects gave written informed consent in accordance with the Declaration of Helsinki. The protocol was approved by the Penn State University’s Institutional Review Board.

## Author Contributions

SC contributed to conception of the work, and collection, cleaning, analysis, and interpretation of data, and she was responsible for drafting and revising the manuscript. NVD contributed to conception of the work, cleaning of physiological data, and revising the manuscript. MM contributed to collection and cleaning of physiological and behavioral data. DA and RA contributed to the cleaning of behavioral data and revising the manuscript. JS supervised the project and contributed to all aspects of the work.

## Conflict of Interest Statement

The authors declare that the research was conducted in the absence of any commercial or financial relationships that could be construed as a potential conflict of interest.

## Footnotes

1 We explored gender differences in self-reported, behavioral (facial expressions), and physiological responses to the disgust film. Self-reported emotions in response to the disgust film did not differ by gender, *p*s > 0.05. Similarly, there were no significant gender differences in facial expressions of disgust, anger, and fear in response to the disgust film, *p*s > 0.05. However, males showed more happiness expressions than females, *t*(55) = −2.35, *p* = 0.023, while females showed more expressions of surprise, *t*(89) = 2.91, *p* = 0.005, and sadness *t*(103) = 2.96, *p* = 0.004, relative to males. Looking at physiological responses, males showed greater SCL responses than females, *t*(81) = −2.44, *p* = 0.017, but there were no other significant gender differences across the remaining physiological indices, *p*s > 0.05. We also examined whether emotional fit differed by gender. There were no gender differences in self-report emotional fit at all three time points, as well as physiological emotional fit at the two available time points, *p*s > 0.05. However, there were significant gender differences in behavioral emotional fit indices at both Times 1 and 2, such that males showed greater behavioral emotional fit than did females, *t*(120) = −2.24, *p* = 0.027, *t*(118) = −2.78, *p* = 0.006, for Times 1 and 2, respectively. Given these gender differences in facial expressions in response to disgust film, as well as in behavioral emotional fit, we included gender as a covariate in the regression models testing the effect of behavioral emotional fit on outcome variables. This did not change any of the reported patterns of results, and therefore these analyses were not included in the manuscript given that examination of gender was outside of the scope of the present study.
